# Serological evidence of rift valley fever virus among acute febrile patients in Southern Mozambique during and after the 2013 heavy rainfall and flooding: implication for the management of febrile illness

**DOI:** 10.1186/s12985-016-0542-2

**Published:** 2016-06-08

**Authors:** Eduardo Samo Gudo, Gabriela Pinto, Jacqueline Weyer, Chantel le Roux, Arcildo Mandlaze, Américo Feriano José, Argentina Muianga, Janusz Tadeusz Paweska

**Affiliations:** National Institute of Health, Ministry of Health, Av Eduardo Mondlane 1008, Ministry of Health Main Building, 2nd floor, PO Box 264, Maputo, Mozambique; Centre for Emerging and Zoonotic Diseases, National Institute of Communicable Disease, of the National Health Laboratory Service, Sandringham, South Africa; Faculty of Health Sciences, School of Pathology, University of Witwatersrand, Johannesburg, South Africa

**Keywords:** Rift valley fever virus, Heavy rainfall, Arbovirus, Sub-Saharan Africa

## Abstract

**Background:**

Rift Valley fever virus (RVFV) remains heavily neglected in humans in Mozambique, even though recent outbreaks were reported in neighboring countries in humans and several cases of RVFV in cattle were reported in several districts in Mozambique.

**Findings:**

We conducted a cross sectional study during and after severe flooding that occurred in 2013 in Mozambique. Paired acute and convalescent serum samples were tested from febrile patients attending a primary health care unit in a suburban area of Maputo city for the presence of IgG and IgM antibodies against Rift Valley fever virus (RVFV) using enzyme-linked immunosorbent assay (ELISA). Seroconversion of IgG anti-RVFV was observed in 5 % (10/200) of convalescent patients and specific IgM anti-RVFV was detected in one acute patient (0.5 %; 1/200). All sera from acute patient tested negative by real time PCR.

**Conclusion:**

In conclusion, our results suggest that RVF represent an important but neglected cause of febrile illness following periods of flooding in southern Mozambique.

## Introduction

Rift Valley Fever Virus (RVFV) is a mosquito borne zoonotic virus that has emerged as an important cause of febrile illness in several sub-Saharan countries [1–5], including those that share their borders with Mozambique, such as Tanzania and South Africa [4, 6–8]. The likelihood that RVF also causes disease in humans in Mozambique is high because of the geographical proximity, environmental similarities and frequent trade between Mozambique and these countries. In Mozambique, RVFV in humans has been poorly studied and only one study was conducted more than 30 years ago among healthy pregnant women and found a RVFV prevalence of 2 % [6]. Since then, no other study was conducted in humans in Mozambique.

Recent publications demonstrated that anti-RVFV IgG was often detected in cattle in Maputo and Gaza Province, both situated in the southern Mozambique [9–11], and in two rural villages in the center of the country [12]. This reinforces the concern that RVFV may be causing undiagnosed disease in humans in Mozambique, particularly in places where recent cases of RVFV were reported in cattle [10–12]. The risk of outbreaks of RVFV in Mozambique is substantial, as the country is ranked the third most vulnerable country in Africa to extreme weather events. Indeed the frequency of floods increased in the last two decades [13] and it is well known that outbreaks of RVFV commonly coincide with periods of heavy rainfall [14, 15]. Lastly, clinical presentation of RVFV resembles that of malaria, leading to misdiagnosis of malaria, mistreatment with anti-malarial drugs [16] and inappropriate management of febrile illness. Given this gap in the literature, we conducted this study with the aim to investigate the burden of RVFV in febrile patients in southern Mozambique during and after the heavy rainfall and flooding that occurred in 2013.

## Methods

### Study design and study setting

**Fig. 1:**
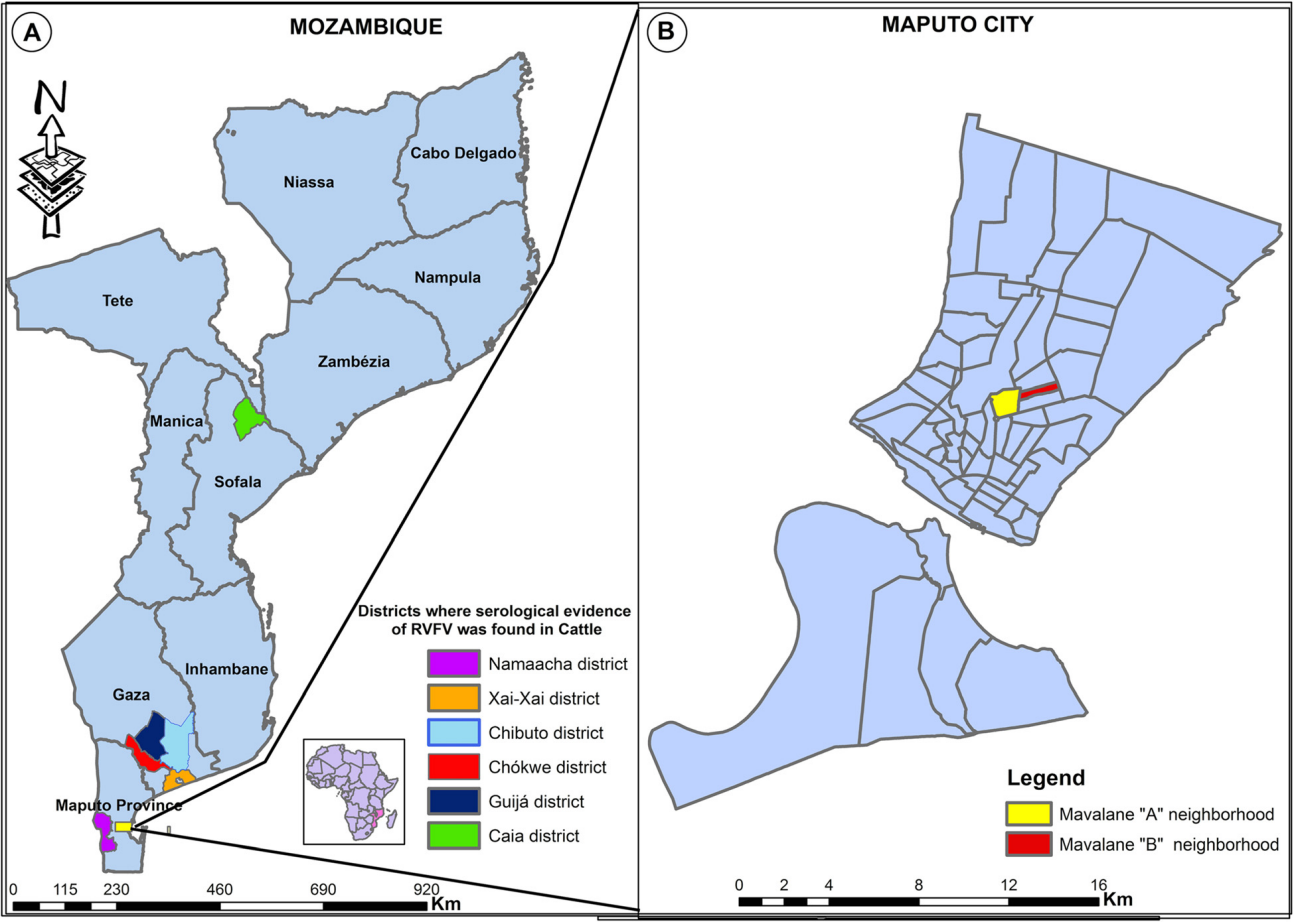
Geographical representation of study area. **a** shows the geographical localization of Mozambique in south east Africa (Mozambique is highlighted in pink in the small map of Africa) and map of Mozambique (larger map). Coloured districts in Mozambique map, represents districts where cases of RVFV were previously reported in Cattle. **b** is the representation of the neighborhood of Mavalane health area covered by the Mavalane health center. Mavalane health area comprises two geographical and well delimited areas, namely, Mavalane “A” (yellow) and Mavalane “B” (red) neighborhood

A cross-sectional study was conducted at the Mavalane Health Center, a primary health care facility situated in southern Mozambique. This health facility serves a large suburban area in Maputo City with a total surface area is 108 km^2^ and a total population of 293 361 habitants (see Fig. 1). The households are precarious and sanitation is poor. In Maputo, the rainy season runs from November through March. The southern region of Mozambique including the study area was heavily affected by intense rainfalls between January and March 2013, which resulted in flooding in several areas. Rainfalls in this period reached up to 300 mm within 10 days.

All patients attending this health facility between January and September 2013 with acute febrile syndrome (axillary temperature > 37.5 °C) and age > 5 years were invited to participate. Written consent was requested prior to enrollment. This study was approved by the National Bioethical Committee in Mozambique (Ref: IRB00002657) prior to initiation. Exclusion criteria included, pregnancy, presence of psychiatric disease and febrile disease with a readily identifiable focus of infection, such as otitis media, sinusitis, purulent pharyngitis, cellulitis, urinary tract infection, dental abscess, septic arthritis, pneumonia or pelvic inflammatory disease.

### Sample collection

A total volume of 10 mL of blood was requested from each volunteer during the enrollment visit (acute sample) and collected into K_3_EDTA tube (5 ml) and into serum separation tube (5 ml, both from BD Vaccutainer, USA). All participants were requested to return to the health facility three weeks later for follow up and collection of additional 5 mL of blood (convalescent sample) into K_3_EDTA tube.

### Questionnaire

A standardized questionnaire was used to collect clinical, demographic and epidemiological data, including information on risk factors for RVFV infection.

### Laboratory testing

#### Blood smear microscopy

Acute sample drawn from each participant was tested for malaria using blood smear microscopy. A thick blood smear was mounted from anticoagulated whole blood. All blood smears were stained using the Giemsa protocol and screened for *Plasmodium falciparum, P.malarie, P. ovale and P. vivax* using light microscopy. Parasite density was estimated by means of a semi-quantitative scale [17].

#### Rift Valley fever virus serology and TaqMan probe-based one-step real-time RT-PCR

Samples were shipped and tested at The Center for Emerging and Zoonotic Diseases of the National Institute for Communicable Diseases, National Health Laboratory Service (NICD/NHLS) in South Africa. The testing algorithm was as follows, at first, all convalescent serum samples were initially tested using recombinant nucleoprotein (rNP)-based anti-RVFV IgG ELISA [18]. If the convalescent sample was positive, the corresponding acute sample was screened using the same test. Patients with evidence of seroconversion for anti-RVFV IgG antibodies, were classified as acute RVFV infection. In order to confirm the presence of acute infection, acute serum samples, from seroconverting patients were screened using anti-RVFV IgM ELISA [19] and for RVFV RNA using TaqMan probe-based one-step real-time RT-PCR [20] targeting the RVFV Gn gene. RNA was extracted from sera using a QIAamp viral RNA mini kit (QIAGEN, Germany) as per manufacturer’s instructions. Acute samples were tested for IgM because our previous research showed that these antibodies were detectable as early as 3-4 days post experimental infection in sheep [19, 21] and 6 days post administration of RVFV vaccine in humans [19]. Previous RVFV exposure was defined as presence of anti-RVFV IgG antibodies, both in the acute and convalescent visit. Negative anti-RVFV infection was defined as an absence of IgG anti-RVFV antibodies in the convalescent serum sample.

For ELISA testing, we followed strictly the instructions described in published literature and details of the testing procedures and interpretation of IgM and IgG assays, are described in the two published manuscripts [18, 19]. The sensitivity and specificity of anti-RVFV IgG ELISA was 99.7 and 99.6 %, respectively and cut off was set at 28.98 percentage of positivity of internal high positive control (PP) [18]. The sensitivity anti-RVFV IgM ELISA was 100 % as early as 4 days post infection and the specificity was 99.6 % and cut off was set at 7.1 PP [19]. PP is calculated using the following formula: (mean net OD of test sample/mean net OD of high-positive control)/100.

### Data analysis

Data analysis was performed using the statistics package STATA 9.0 (College Station, Texas: StataCorp, USA, 2005). Simple frequencies were calculated for each study variable. Study groups were compared using Kruskal Wallis test. Associations between categorical variables were determined using logistic regression analysis. A *p* value < 0.05 was considered of statistical significance.

## Results

**Fig. 2:**
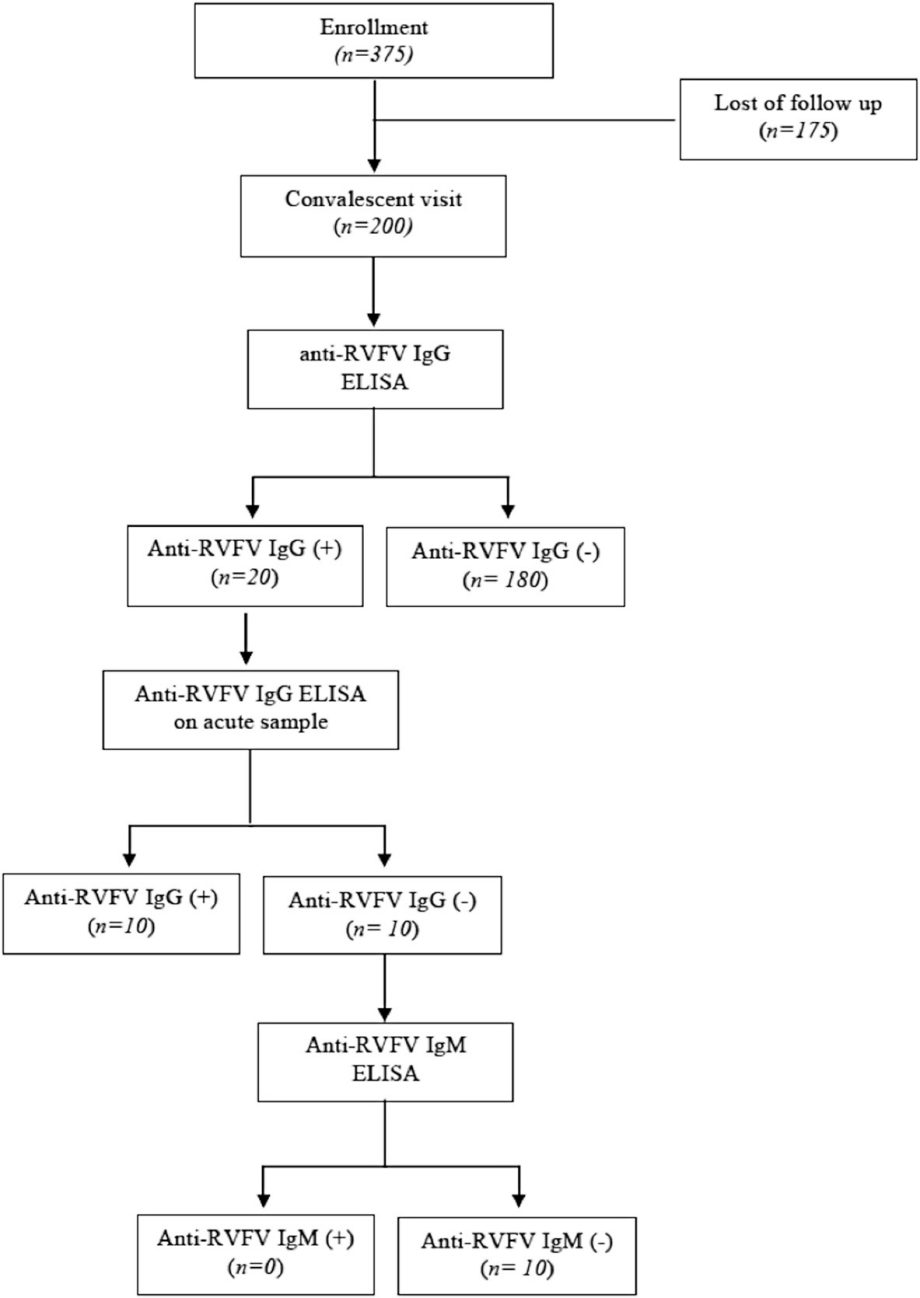
Participant’s recruitment and sample testing. Out of 375 patients recruited, 200 returned to the convalescent visit of which 20 were positive (10.0 %). The corresponding 20 acute samples of those patients were screened using anti-RVFV IgG ELISA. Evidence of seroconversion could be demonstrated in 10 (5.0 %) out of 20 of those patients. Amongst 10 patients who seroconverted for IgG anti-RVFV, only one tested positive the presence of anti-RVFV IgM antibody

Three hundred and seventy five patients were enrolled between January and September 2013 and 175 did not return to their convalescent visit (see Fig. 2), although efforts were undertaken by the research team to reach them by phone a few days prior to the expected date of convalescent appointment. Of note, the average number of days between onset of fever and recruitment and convalescent visit were 1 day and 25 days, respectively.

The median age of study participants was 28 years (IQR: 21-36 years) and 56.7 % (98/173) were female.

Of the 200 convalescent samples, 10 % (20/200) tested positive for IgG anti-RVFV. Seroconversion for IgG anti-RVFV was confirmed in 10 (5.0 %, 10/200) samples. Most of samples from patients with serological evidence of acute infections (defined as presence of seroconversion of IgG anti-RVFV antibodies between acute and convalescent sample), were clustered between February and April with a peak in April (see Table 1). The corresponding acute sample from seroconverting patients, were additionally tested for the presence of IgM anti-RVFV and one (0.5 %, 1/200) was positive. In term of diagnostic and therapeutic implications, 6 out of 9 (67 %) patients who met the case definition for acute RVFV infection were misdiagnosed as malaria and treated with anti malarial medication (see Table 1).

**Table 1:** Chronological information and laboratory results of the 10 patients with serological evidence of acute RVFV infection

#	Date of onset of symptoms	Date of blood collection	# days	Seroconversion of anti-RVFV IgG antibodies^a^	IgM anti-RVFV in the acute sample	Real- time PCR for RVFV	Malaria blood smear	Anti-malarial treatment
1	Feb 23, 2013	Feb 24, 2013	1	yes	-	-	-	Yes
2	Feb 26, 2013	Feb 27, 2013	1	yes	-	-	-	Yes
3	Apr 17, 2013	Apr 18, 2013	1	yes	-	-	-	No
4	Apr 23, 2013	Apr 23, 2013	<1	yes	-	-	-	Yes
5	Apr 26, 2013	Apr 26, 2013	<1	yes	-	-	-	Yes
6	Apr 27, 2013	Apr 27, 2013	<1	yes	-	-	-	No
7	Apr 27, 2013	Apr 29, 2013	2	yes	+	-	-	Yes
8	May 10, 2013	May 11, 2013	1	yes	-	-	-	NA
9	May 11, 2013	May 12, 2013	1	yes	-	-	-	Yes
10	July 17, 2013	July 19, 2013	2	yes	-	-	-	No

#days: between onset disease and sample collection, *np* not performed, *NA* not available

^a^Seroconversion is defined as IgG anti-RVFV level below ELISA cut-off in the acute serum and above ELISA cut-off in convalescent serum

Real-time PCR was performed on sera from 10 patients with serological evidence of acute RVFV infection and all were negative.

A total of 36 out of 375 patients enrolled were positive for malaria, yielding a prevalence rate of 9.6 %. Co-infection with RVFV and malaria were not found among those patients.

Based on the serological profile, patients were stratified into three main groups, namely i) acute RVFV infection if they seroconverted for anti-RVFV IgG antibodies between the acute and convalescent visit (10/200, 5.0 %), ii) previous RVFV exposure if they presented anti-RVFV in both the acute and convalescent visit (10/200, 5.0 %) and iii) no anti-RVFV antibodies (180/200, 90.0 %). Table 2 shows that the three groups were similar in term of age, gender and clinical presentation. Chills and abdominal pain were the most common clinical presentation. No severe case was reported and no patients presented any form of hemorrhage, neurological sign or blindness

**Table 2:** Clinical and demographic characteristics of study participants stratified by RVFV infection status

Socio-demographic characteristics	Acute RVFV Infection	Previous RVFV exposure	No RVFV antibodies	*p-*value
	n (%)	n (%)	n (%)	
Total	*n* = 10	*n* = 10	*n* = 190	
Age				
Median	30	23	28	0.202
IQR	19-42	19-30	21-35	
Gender				
Male	6 (60.0)	5 (50.0)	80 (44.4)	0.603
Female	4 (40.0)	5 (50.0)	100 (55.6)	
Recent contact with cattle	5 (50.0)	4 (40.0)	51 (28.3)	0.136
Recent international travel	1 (10.0)	0	12 (6.7)	0.636
Chills	8 (80.0)	7 (70.0)	106 (58.9)	0.339
Pruritus	1 (10.0)	0	19 (10.6)	0.556
Headache	9 (90.0)	8 (80.0)	131 (73.2)	0.454
Arthralgia	5 (55.6)	17 (38.6)	46 (30.1)	0.192
Skin Rash	0	1 (10.0)	47 (8.9)	0.609
Myalgia	5 (50.0)	6 (60.0)	77 (42.7)	0.524
Abdominal pain	6 (60.0)	7 (70.0)	81 (45.0)	0.213
Vomiting	2 (20.0)	1 (10.0)	131 (17.2)	0.812
Diarrhea	2 (20.0)	2 (20.0)	21 (11.7)	0.872
Hemorrhage	0	0	0	-

## Discussion

To our knowledge this is the first investigation of RVFV among febrile patients in Mozambique and we found that 5 % (10/200) of febrile patients seroconverted to IgG antibodies against RVFV between their acute and convalescent samples. Although novel for Mozambique, this finding is not surprising, based on the following arguments: i) A recent study conducted by our group in the same area showed that *Aedes* mosquitoes were abundant [22] ii) recent studies conducted in several districts in the surroundings of the study area found elevated frequencies of anti-RVFV IgG antibodies in cattle [10, 11], iii) several outbreaks and sporadic cases have been reported in countries neighboring Mozambique such as South Africa and Tanzania [4, 8, 23], iv) patients were recruited during and after the severe heavy rainfalls that occurred in southern Mozambique in 2013 [8, 15], v) people living in the study area travel regularly to the surroundings districts where elevated frequencies of antibodies were recently documented in cattle [10, 11] and vi) cattle population is much more frequent than sheep in the study area [24]. Although the source of infection was not investigated in this study, the fact that most of cases occurred between February through May, peaking in April, suggest that heavy rainfall and floods that occurred in the same period might have influenced the occurrence of RVFV in Mozambique. No entomological or livestock investigation was performed as this was an exploratory study. We acknowledge that lack of this information is a limitation. We plan to incorporate these components into a forthcoming investigation.

Only 1 patient was positive for anti-RVFV IgM antibodies in the acute sample. This may be explained by the fact that the average number of days between onset of fever and blood collection was one day, and two patients were enrolled two days post onset of fever, likely, before the seroconversion of IgM antibodies. IgM anti-RVFV was not performed in the convalescent samples and we acknowledge this as a limitation of our study, but the demonstrated seroconversion for IgG antibodies against RVFV should be regarded as evidence for recent exposure to RVFV. The anti-RVFV IgG ELISA was intensively validated according to the results of a virus neutralization test in 2967 sera collected in Africa and was shown to have diagnostic sensitivity and sensitivity close to 100 % [18, 19].

PCR results were all negative. Negative PCR results might be attributed to the fact that viremia in these patients is transient. We also argue that PCR results might be explained by the fact that serum samples were poorly handled, by repetitive and multiple freeze-thaw cycles and long periods of storage at room temperature, resulting in degradation of the RNA or viral particles.

No cases of severe diseases was reported and this might be explained by the fact that patients were recruited at the outpatient triage at the primary health care facility, where the majority of patients presents with non-severe disease.

Of remark, our data showed that 67 % of patients with serological evidence of acute RVFV infection and negative malaria blood smear were treated with antimalarial drugs. This demonstrates that most of RVFV infections are misdiagnosed as malaria.

## Conclusion

In conclusion, our data suggests that RVFV infection occurs among febrile patients in the periods of heavy rainfalls and flooding in southern Mozambique and that most of cases are misdiagnosed as malaria, leading to mistreatment with antimalarial drugs.
